# Poly(2‐alkyl‐2‐oxazoline)‐Heparin Hydrogels—Expanding the Physicochemical Parameter Space of Biohybrid Materials

**DOI:** 10.1002/adhm.202101327

**Published:** 2021-09-20

**Authors:** Dominik Hahn, Jannick M. Sonntag, Steffen Lück, Manfred F. Maitz, Uwe Freudenberg, Rainer Jordan, Carsten Werner

**Affiliations:** ^1^ Leibniz Institute of Polymer Research Dresden Max‐Bergmann Center of Biomaterials Dresden Hohe Str. 6 01069 Dresden Germany; ^2^ Dresden Initiative for Bioactive Interfaces & Materials Technische Universität Dresden Mommsenstr. 4 01069 Dresden Germany; ^3^ Professur für Makromolekulare Chemie Faculty of Chemistry and Food Chemistry Technische Universität Dresden Mommsenstr. 4 01069 Dresden Germany; ^4^ Leibniz Institute of Polymer Research Dresden Max‐Bergmann Center of Biomaterials Dresden Hohe Str. 6 01069 Dresden Germany; ^5^ Center for Regenerative Therapies Dresden (CRTD) Fetscherstr. 105 01307 Dresden Germany

**Keywords:** heparin, hydrogels, poly(2‐alkyl‐2‐oxazolines), thermoresponsiveness

## Abstract

Poly(ethylene glycol) (PEG)‐glycosaminoglycan (GAG) hydrogel networks are established as very versatile biomaterials. Herein, the synthetic gel component of the biohybrid materials is systematically varied by combining different poly(2‐alkyl‐2‐oxazolines) (POx) with heparin applying a Michael‐type addition crosslinking scheme: POx of gradated hydrophilicity and temperature‐responsiveness provides polymer networks of distinctly different stiffness and swelling. Adjusting the mechanical properties and the GAG concentration of the gels to similar values allows for modulating the release of GAG‐binding growth factors (VEGF165 and PDGF‐BB) by the choice of the POx and its temperature‐dependent conformation. Adsorption of fibronectin, growth of fibroblasts, and bacterial adhesion scale with the hydrophobicity of the gel‐incorporated POx. In vitro hemocompatibility tests with freshly drawn human whole blood show advantages of POx‐based gels compared to the PEG‐based reference materials. Biohybrid POx hydrogels can therefore enable biomedical technologies requiring GAG‐based materials with customized and switchable physicochemical characteristics.

## Graphical abstract

Hydrogels made of different poly(2‐alkyl‐2‐oxazolines) (POx), crosslinked with the glycosaminoglycan (GAG) heparin, are shown to provide distinctively different swelling and temperature responsiveness, allowing for a temperature‐controlled release of GAG‐binding signaling molecules and a far‐reaching modulation of further cell‐instructive properties. The newly established biohybrid POx‐GAG hydrogel platform is therefore envisioned to enable various advanced biomedical technologies.

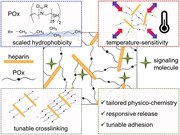

## 1 Introduction

Hydrogels, networks of highly hydrated polymers, are widely used in well‐established biomedical applications, including membranes for blood purification, intraocular and contact lenses, drug delivery systems, and coatings of cardiovascular catheters.^[^ ^1^ ^]^ Moreover, cell‐instructive hydrogels are critical enablers in emerging regenerative therapies and in vitro tissue/disease models.^[^ ^2^ ^]^

As a particularly powerful class of hydrogel materials, we and others have recently reported biohybrid gel systems containing glycosaminoglycans (GAG). These systems are able to complex, stabilize, and sustainably deliver a plethora of important biomolecular effectors^[^ ^3^ ^]^ and, thus, are customizable as biomimetic matrix templates in various different approaches.^[^ ^2^, ^4^ ^]^ Heparin and selectively desulfated heparin derivatives were covalently crosslinked with multiarmed poly(ethylene glycol) (starPEG) by active ester chemistry or—for the in situ assembly of the materials in a target tissue or for embedding of cells—by a cytocompatible Michael‐type addition reaction of thiol‐functionalized starPEG to maleimide‐conjugated GAG groups.^[^ ^5^ ^]^ In both reaction schemes, the degree of functionalization and the molar ratio of the polymeric gel precursors, as well as the solid content of the reaction mixture allow for “programming” the network properties of the resulting materials independent of the concentration of the GAG units in a theoretically predicted manner.^[^ ^5^, ^6^ ^]^ The incorporation of enzymatically cleavable peptide units and the conjugation of adhesion receptor ligand peptides provided additional options for producing multifunctional materials that recapitulate basic features of extracellular matrices in a liberal and simplistic system.^[^ ^4^, ^7^ ^]^ Beyond that, our starPEG‐GAG hydrogel toolbox was successfully adapted as a coagulation‐enzyme‐controlled anticoagulant delivery system that effectively prevents the coagulation of human whole blood in the absence of additional anticoagulants.^[^ ^8^ ^]^

For adjusting the functional properties of the elaborated starPEG‐GAG gel materials, the choice of the polymeric precursors was used as an obvious free parameter,^[^ ^6^, ^9^ ^]^ in addition to the control of crosslinking and the incorporation of biomolecular (peptide) units. However, as of now, all these previously published gel systems relied on starPEG units as the synthetic building block. While the nonadhesive, flexible characteristics of PEG proved to be very valuable in many elaborated systems, the limited variability of the characteristics of this component limits the physicochemical tunability of the obtained gel materials. Moreover, concerns raised against the widespread use of PEG, potentially resulting in recognition by and—in rare cases—activation of the immune system.^[^ ^10^ ^]^ The potential hydrolytic cleavage in biological ambiance by the presence of metal ions additionally motivates the search for alternative polymer systems.^[^ ^11^ ^]^

Poly(2‐alkyl‐2‐oxazolines) (POx), recently developed as an alternative to PEG in biomedical applications.^[^ ^12^ ^]^ Presumably since there is no wide use in cosmetics yet, the prevalence of antibodies against these polymers is lower than against PEG. The tertiary amine group in the backbone is more resistant against hydrolysis than the ether group of PEG; further, the alkyl side chain allows versatile functionalization. POx already proved high versatility for hydrogel formation.^[^ ^13^ ^]^ Functionalization of the side groups with adhesive peptides in the hydrogel supports cell adhesion and 3D cell culture and demonstrates the general biocompatibility of this class of polymer.^[^ ^14^ ^]^ End‐group and side‐chain functionalizations with appropriate crosslinkers have been suggested for advanced photocrosslinking or for biomedical injectable hydrogels with in situ crosslinking.^[^ ^15^ ^]^ Cationic‐functionalized POx‐based hydrogels have been further discussed as delivery systems for electrostatically bound DNA with the release triggered by heparin.^[^ ^16^ ^]^

The variability of the 2‐alkyl chain of POx, obtained by living cationic ring opening polymerization of various monomers including 2‐methyl‐, 2‐ethyl‐, 2‐iso‐propyl‐, and 2‐n‐propyl‐2‐oxazoline results in polymers that differ in solubility from hydrophilic to hydrophobic and can exhibit thermoresponsiveness or amphiphilic characteristics.^[^ ^12^, ^17^ ^]^ Further, the controlled/living polymerization technique enables the introduction of nucleophilic termination reagents as end‐functionalities with a high degree of functionalization.^[^ ^18^ ^]^

This study, therefore, explored these alternative synthetic polymer units of variable physicochemical properties instead of the starPEG units in the design of GAG‐containing biohybrid hydrogels. Thiol‐terminated poly(2‐methyl‐, 2‐ethyl‐, 2‐iso‐propyl‐, and 2‐*n*‐propyloxazolines) were synthesized and crosslinked with differently maleimide‐conjugated heparin units to adjust the crosslinking degree. Since linear POx units were used, a nonbranched thiol‐terminated PEG similarly containing about 50 repeating units (as all POx variants) was included in the study as a reference. The obtained hydrogels were thoroughly examined for their mechanical properties and thermoresponsiveness. Beyond that, we analyzed the impact of the POx type (and its temperature‐induced conformation) on the uptake and release characteristics of the gel materials for selected growth factors, investigated the adsorption of fibronectin and the adhesion of fibroblasts and bacteria onto the surfaces of the compared gel materials, and assessed their hemocompatibility.

## 2 Results and Discussion

### 2.1 Synthesis of Thiol‐Terminated Poly(2‐alkyl‐2‐oxazolines)

**Figure 1:**
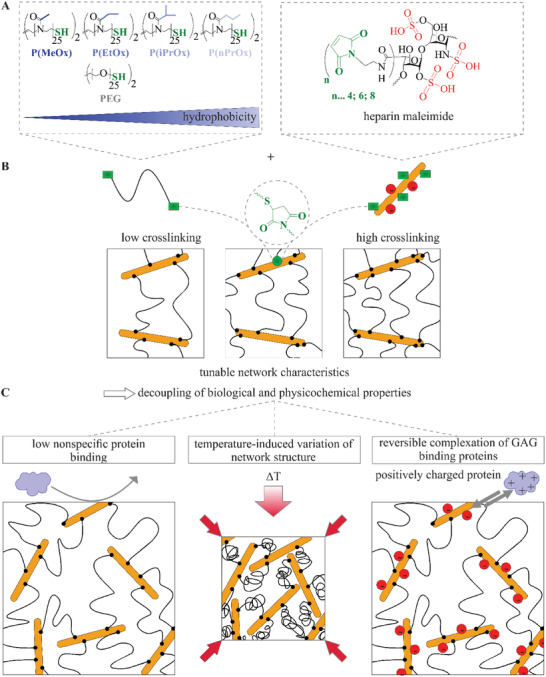
Formation of the compared POx‐HEP and PEG‐HEP gel types. A) Chemical structures of the POx, PEG, and heparin units. B) Network structure resulting from the reactive conversion of POx (or PEG) dithiol with HEP‐maleimide by Michael‐type addition, the crosslinking degree can be adjusted by the maleimidation of heparin and by the concentration of the synthetic polymer. C) Biofunctional key properties of the developed materials.

POx dithiols of gradated hydrophobicity (**Figure** **1**A) were synthesized by living cationic ring opening polymerization. For providing two propagating chain ends, initiation of the living cationic ring opening polymerization was achieved using the well‐studied *trans‐1,4‐dibromobut‐2‐ene*.^[^ ^19^ ^]^ The analysis showed that under these conditions for two termination groups, a reliable chain length was achieved with a maximum of ≈25 repetition units per side (50 in total). Longer polymers resulted in multimodal polymeric distributions due to premature polymerization termination, cyclization, and chain–chain termination. From the analytical data, a low polydispersity (PDI) of about 1.1, comparable number of repetition units, and sufficient functionalization degree of the polymer after polymerization (endgroup fidelity, determined by ^1^H NMR, see Figure S1, Supporting Information) of about 70–90% (**Table** **1**) were received. Higher chain length up to 80 repetition units, but substantially higher PDI values close to 2, have been reported for increased reaction temperature to 100 °C for over 5 h and alternating the solvent to benzonitrile.^[^ ^20^ ^]^

**Table 1:** Analytical data of poly(2‐alkyl‐2‐oxazolines); Abbreviations: poly(2‐methyl‐2‐oxazoline)—P(MeOx); poly(2‐ethyl‐2‐oxazoline)—P(EtOx); poly(2‐iso‐propyl‐2‐oxazoline)—P(iPrOx); poly(2‐*n*‐propyl‐2‐oxazoline)—P(nPrOx); degree of polymerization—DP*_n_*; number average molar mass—M*_n_*; mass average molar mass—M_w_; nuclear magnetic resonance spectroscopy—NMR (see Figure S1, Supporting Information); size exclusion chromatography—SEC (see Figure S3, Supporting Information). Endgroup fidelity: functionalization of the polymer after polymerization, determined by ^1^H NMR (signal overlay prevented determination for P(EtOx) and P(nPrOx)); thiol group reactivity: finally achieved endgroup number available after deprotection (determined by Ellman's); active thiol groups per polymer determined from the thiol group reactivity

Polymer	DP*_n_* ^theo^ [M]_0_/[I]_0_	*M* ^theo^ [g mol^−1^]	*M_n_* ^NMR^ [g mol^−1^]	*M*_w_^SEC^ [g mol^−1^]	PDI (*M*_w_/*M_n_*)	Endgroup fidelity [%]	Thiol group reactivity [%]	Active thiol groups per polymer
P(MeOx)	49	4372	4712	4401	1.17	72	77	1.54
P(EtOx)	50	5157	5653	5720	1.15	‐	91	1.88
P(nPrOx)	50	5858	6311	5085	1.15	‐	88	1.76
P(iPrOx)	50	5858	5066	5130	1.08	87	89	1.78
PEG‐SH	50	2450	‐	‐	‐	‐	89	1.78

Since the reactivity of the terminating thiols is essential for reproducible gel formation using the Michael‐type addition reaction and since the thiol moiety is highly susceptible to oxidation,^[^ ^21^ ^]^ the termination of the polymerization had to be carried out with potassium thioacetate resulting in a protected thiol group precursor, following the protocol by Juang et al.^[^ ^22^ ^]^ The acetate group can be removed by ester hydrolysis^[^ ^23^ ^]^ under acidic or basic conditions. For monofunctionalized acetyl thiols, sodium or potassium hydroxide in ethanol/water mixture is typically used, gaining rapid quantitative hydrolysis, resulting in a sodium/potassium thiolate. Applying Ellman's reagent for thiol group quantification resulted in a very low thiol reactivity of the POx dithiols after deprotection with KOH/NaOH. For this reason, different hydrolyzing agents with increasing p*K*_A_ values, from highly alkaline to highly acidic, were tested (results shown in Figure S4, Supporting Information). The highest thiol reactivity was achieved by hydrolysis in aqueous ammonium hydroxide solution diluted with methanol. This approach offered several advantages, as ammonium hydroxide is a mild base, strong enough to hydrolyze the thioester, but not strong enough to amplify thiol group oxidation. Additionally, both methanol and ammonium hydroxide are volatile and thus can be quantitatively removed under vigorous inert gas flow, resulting in ammonium thiolate. Subsequently, the oxidation was quenched using *tris*(2‐carboxyethyl)phosphin (TCEP), a mild, effective disulfide bond reducing agent, which was removed by the subsequent dialysis steps to avoid cytotoxicity. With the described method, all used polymers reached sufficient thiol group reactivity (determined by Ellman's) comparable to the end‐group fidelity determined by ^1^H NMR (Table 1).

### 2.2 Formation and Mechanical Characterization of POx‐HEP Hydrogels

Binary hydrogels of PEG or POx and HEP were synthesized by Michael‐type addition of thiol‐terminated PEG or POx to HEP‐maleimide. The maleimide group was coupled to HEP using 1‐ethyl‐3‐(3‐dimethylaminopropyl)carbodiimid (EDC)/*N*‐hydroxysuccinimide (NHS) conjugation chemistry as shown before^[^ ^7^ ^]^ (chemical structure shown in Figure 1A). While this scheme has been previously established and widely applied for thiol(ated peptide)‐terminated starPEG and HEP‐maleimide, no previous study used POx (nor linear PEG) as the synthetic component. For comparison of the obtained gel characteristics, we herein used the synthesized thiol‐terminated POx (see above) of about 50 repeating units in total and a linear PEG dithiol with a comparable number of repeating units. A (four‐arm) starPEG thiol with a comparable number of repeating units, as previously used in the preparation of starPEG‐HEP materials, was further included to connect this study to our earlier work. For gel preparation, the ice‐cold precursor solutions were mixed and cast at room temperature, except for P(nPrOx)‐HEP hydrogels, which had to be kept ice‐cooled to prevent displacement effects from polymer mixing. With a careful adjustment of the number of thiol groups (Table 1) to the number of maleimide groups per heparin, the crosslinking degree can be tuned to achieve different network densities resulting in gradually adjusted physical properties (see Figure 1B). The quantitative turnover was indicated as the stiffness of the gels and was observed to be maximal for stoichiometric availability of thiol and maleimide groups (Figure S5C, Supporting Information). Gels containing linear PEG instead of starPEG were found to exhibit a slightly lower stiffness (see Figure S5A, Supporting Information). The Michael‐type addition reaction applied for gel formation is very rapid and quantitative in all cases, i.e., it effectively avoids the formation of side products and thus the necessity of hydrogel purification.^[^ ^7^ ^]^

In sum, the far‐reaching gradation of POx derivatives was expected to customize functional biohybrid materials when crosslinked with the protein‐complexing GAG heparin (Figure 1C). Five different synthetic polymers were chosen, the mechanical properties, swelling, and hydrophobicity of the obtained hydrogels were assessed.

**Figure 2:**
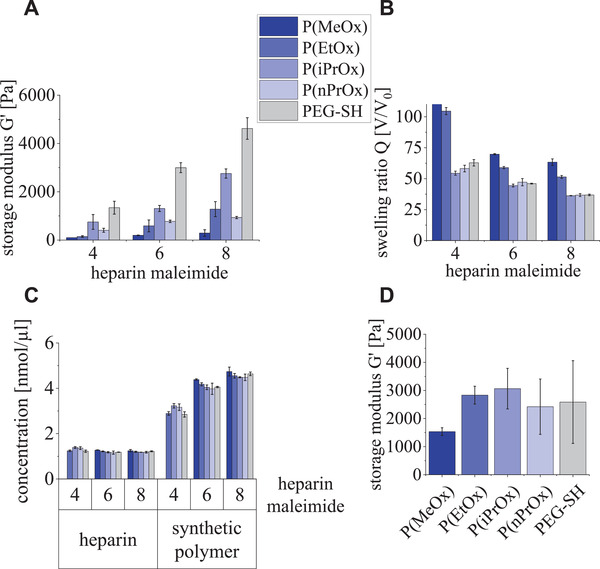
Mechanical properties of POx‐HEP and PEG‐HEP gels. A) Storage moduli of gels with differing crosslinking degrees and constant solid content of 5% (determined at room temperature using rheometry). B) Swelling degrees *Q* (gel volume after equilibrium swelling compared to dried gel volume) of samples as in (A). C) Concentrations of both HEP and the synthetic polymer component in the respective swollen hydrogel type dependent on the crosslinking degree as determined by the HEP‐maleimide functionalization (all concentrations were determined in hydrogels with 10% solid content). D) Storage moduli of gels at constant heparin concentration of 4.5 × 10^−3^ m and adjusted hydrogel parameters as in Table 2 (determined at room temperature using rheometry).

The crosslinking degree of the hydrogels was adjusted by the molar ratio of the reactive precursors (POx/PEG and HEP‐maleimide) and their degree of functionalization (i.e., the choice of HEP‐maleimide carrying 4, 6, or 8 equivalents of maleimide). Since the maleimide‐thiol‐reaction is nearly quantitative, the number of reactive maleimide moieties determines the crosslinking degree of the hydrogels whose increase manifests in increasing storage moduli (*G*’, **Figure** **2**A), as determined by parallel plate rheometry and nanoindentation measurements using atomic force microscopy (AFM), and decreasing swelling degrees (Figure 2B) at comparable solid contents of the reaction mixture, as previously established for starPEG‐HEP gels.^[^ ^7^, ^24^ ^]^

Comparing the different POx‐based hydrogels showed an increase in the stiffness with increasing side chain length (and resulting hydrophobicity) of the POx (from MeOx to iPrOx) except for nPrOx (see Figure 2A). The deviating effect of nPrOx was attributed to the even higher hydrophobicity of this repeating unit.^[^ ^12^, ^17^ ^]^ Elevated hydrophobicity exceeding a certain level may restrict the miscibility of the POx with heparin resulting in network defects. Using organic solvents or lower salt contents might overcome this problem; however, it was not tested here since these conditions would not be compatible with the incorporation of proteins or cells.^[^ ^4d^ ^]^ Likewise, POx‐types with longer side chains reduce the swelling for hydrogels of a given crosslinking degree (see Figure 2B); however, nPrOx and iPrOx surprisingly showed similar swelling degrees despite the observed difference in storage moduli. This effect might be caused by the higher hydrophobicity of nPrOx, which restricts the water uptake of the network, even though it is less dense.

The PEG‐HEP gel was found to be stiffer than all compared POx‐HEP gels, which can be attributed to the lower monomer mass of ethylene glycol (44 g mol^−1^) in comparison with the oxazolines (85–113 g mol^−1^): Since the solid content of the reaction mixture was kept constant at 5%, the PEG‐HEP gels contained a higher number of the two building blocks per volume. For gels formed at a solid content of 10% (i.e., approximately similar number of building blocks as for PEG), P(EtOx)‐HEP (chosen since P(EtOx) is similar hydrophilic as PEG) showed a higher stiffness compared to PEG‐HEP gels (Figure S5B, Supporting Information), which might be explained by differences in chain flexibility—the shorter persistence length of PEG (3.8 Å)^[^ ^25^ ^]^ as compared to P(EtOx) (20 Å)^[^ ^26^ ^]^ can be assumed to result in more flexible chains and thus softer gels—but also to differences in the number of network defects—the lower solid content of PEG‐HEP hydrogels may have resulted in a higher number of defect structures.

These findings were used as guiding principles for adjusting the stiffness and heparin concentration in the biohybrid gels in further experiments (Figure 2C): A set of hydrogels of similar stiffness in the range of 2500–3000 Pa (except for P(MeOx) with 1500 Pa) and a constant heparin concentration of 4.5 × 10^−3^ m (see **Table** **2** and Figure 2D) were used throughout the subsequently reported experiments to unambiguously conclude on the impact of the varying physicochemical characteristics of the synthetic polymer on the resulting gel characteristics and thus to decouple the GAG‐related network effects.

**Table 2:** Gelation parameters for gels of constant heparin concentration (4.5 × 10^−3^ m)

Synthetic polymer	*n* of maleimide per heparin	Solid content [%]	Mean stiffness [Pa]
P(MeOx)	8	13	1530 ± 140
P(EtOx)	4	11	2830 ± 310
P(iPrOx)	4	11	3310 ± 980
Pn(PrOx)	4	10	2420 ± 980
PEG‐SH	4	8	2590±1470

### 2.3 Physicochemical Properties of POx and POx‐HEP Hydrogels

**Figure 3:**
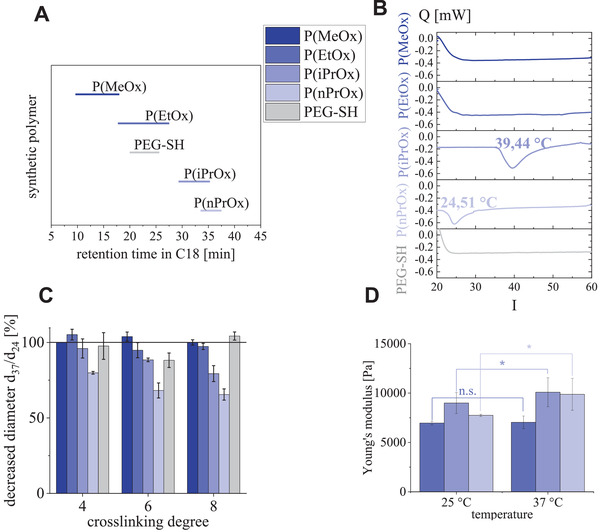
Physicochemical properties of soluble POx and PEG, and of POx‐HEP and PEG‐HEP hydrogels. A) Retention time of dissolved POx and PEG in C18 reverse phase chromatography, using a linear AcN gradient. B) Calorimetric curves in the range of 20–60 °C for POx and PEG in aqueous solution (PBS; 1 mg mL^−1^ polymer concentration adjusted in the solution and in the hydrogel; transition temperatures highlighted). C) Collapsed diameter of hydrogel disks upon transition from room temperature to 37 °C. D) Stiffness of the hydrogels (Young's Modulus corresponding to ≈3 × *G’* assuming a Poison ratio of 0.5) measured at room temperature and at 37 °C by AFM (*p* < 0.001) of nonresponsive P(EtOx)‐HEP and temperature‐responsive P(iPrOx)‐ and P(nPrOx)‐HEP hydrogels.

Poly(2‐alkyl‐2‐oxazolines) offer a broad range of physicochemical properties, reaching from water‐insoluble to highly hydrophilic variants.^[^ ^17b^ ^]^ For homopolymers, it is known that the hydrophobicity increases with the length of the side chain. To characterize the POx used in this study, we analyzed the solution behavior in water by affinity chromatography with a reverse‐phase separation. A linear gradient of AcN in water was utilized to separate the polymers by weakening the hydrophobic interactions of the POx with an alkane‐coated solid‐phase column (C18). We found that the hydrophobicity of the used polymers increases in the following order: P(MeOx) < PEG = P(EtOx) < P(iPrOx) < P(nPrOx) (**Figure** **3**A). Viegas et al.^[^ ^12a^ ^]^ used a similar method for comparing POx and PEG of different molecular weights and reported similar trends.

POx of increased side chain length are known to show a lower critical solution temperature (LCST), i.e., undergo a discrete temperature‐induced phase transition.^[^ ^17b^ ^]^ Differential scanning calorimetry (DSC) was used to determine the related behavior in aqueous solutions (calorimetric curves in Figure 3B). For P(MeOx), P(EtOx), and PEG, the initial decrease of heat flux was regarded as an artifact caused by the instrumental temperature adjustment. For none of these three polymers, a reproducible change in heat flux was observed between 20 and 60 °C accordingly. For P(iPrOx) and P(nPrOx), we found LCST behavior at 39 and 24 °C, respectively, which is in agreement with published data.^[^ ^12b^ ^]^

POx‐HEP hydrogels containing the temperature‐sensitive polymers P(iPrOx) and P(nPrOx) showed a much broader peak in the DSC compared to the response of the plain POx in solution, and the response temperature was shifted to higher values (**Table** **3** and Figure S6, Supporting Information). The broadening of the peak and the shift in the temperature were concluded to result from a disturbed polymer collapse due to steric restrictions of the crosslinked POx‐chains within the network. Temperature‐sensitive hydrogels are known to respond by shrinking of the polymer network at temperature changes, which is designated as network collapse.^[^ ^26b^ ^]^ The equilibrium shrinking degree was quantified as a ratio of the hydrogel disk diameter at physiological temperature (37 °C) compared to the diameter at room temperature as determined by light microscopy calibrated using scale paper (Figure 3C). While the nonresponsive hydrogels containing P(MeOx), P(EtOx), or PEG did not show any shape change, the responsive gels made from P(iPrOx) or P(nPrOx) showed a shrinking in dependence on their respective crosslinking degree. A stronger response was observed for a given POx at higher crosslinking degrees. The maximal shrinking to ≈80 % of the diameter at room temperature was found for gels containing P(iPrOx) and to ≈60 % for gels containing P(nPrOx), at the highest crosslinking degree, respectively. In comparison to pure poly(*N*‐isopropylacrylamide) (PNIPAAM) and P(EtOx) hydrogels,^[^ ^27^ ^]^ all observed shrinkage effects are rather low, probably due to the hindered POx collapse when incorporated in binary hydrogel networks with heparin, as a consequence of repulsive forces in between the latter component and steric restriction of the POx chains inside the hydrogel network, resulting from elastic retraction forces. Despite the rather low shrinking, the gel network is more densely packed at elevated temperatures, which manifests itself in a higher stiffness of the hydrogels, known as thermo‐toughening effect (Figure 3D; elastic moduli determined by AFM: P(EtOx)‐HEP (0.08 kPa), P(iPrOx)‐HEP (1.1 kPa), and P(nPrOx)‐HEP (2.13 kPa)). The observed stiffness increase is comparable to the toughening behavior of hydrogels made of PNIPAAM.^[^ ^28^ ^]^ An influence on the gel structure may also occur upon temperature change for thermoresponsive hydrogels, firmly immobilized to a solid substrate, similar to a system analyzed by Benetti et al.^[^ ^29^ ^]^

**Table 3:** Transition temperatures [°C] of POx and PEG in solution, and of POx‐HEP and PEG‐HEP hydrogels

	Transition temperature in solution	Transition temperature in hydrogels
P(MeOx), P(EtOx), PEG	None	None
P(iPrOx)	39	49
P(nPrOx)	25	30

### 2.4 Growth Factor Release from POx‐HEP Hydrogels

GAG‐based hydrogels are highly beneficial due to their biomimetic administration of soluble signaling molecules, i.e., their capacity to bind, stabilize, and sustainably deliver growth factors and other soluble signaling molecules due to electrostatic interactions between the highly negatively charged GAGs and positively charged domains of the proteins.^[^ ^4^, ^6^, ^30^ ^]^ The sulfation pattern of the GAG‐component and the network characteristics of the PEG‐containing hydrogels were recently demonstrated to modulate these interactions effectively.^[^ ^3^, ^4^, ^6^ ^]^ However, no previous study adjusted the interaction of GAG‐based biohybrid hydrogels to soluble signaling molecules through the variation of the synthetic polymer building block to tune affinity to proteins beyond charge‐driven interactions. For that aim, we formed the above‐described set of POx‐HEP (and PEG‐HEP‐control) hydrogels in presence of the two GAG‐affine growth factors (GFs) VEGF165 and PDGF‐BB (prior to gel formation GFs have been premixed with heparin in a molar ratio of GF:heparin = 1:500 to ensure homogenous distribution and complete incorporation of the proteins within the polymer networks). The hydrogels displayed comparable network structure (stiffness and GAG concentration, Figure 2C,D) at room temperature (but deviating properties at 37 °C for the POx‐containing gels due to the thermal effects, Figure 3). The release of these proteins was then tested at room temperature or 37 °C.

Vascular endothelial growth factor (VEGF165) plays an important role in angiogenesis, bone formation, hematopoiesis, and wound healing,^[^ ^31^ ^]^ while platelet‐derived growth factor (PDGF‐BB) triggers chemotaxis of cells and is of importance in embryogenesis and wound healing.^[^ ^32^ ^]^ Both proteins significantly differ in their charge patterns as well as hydrophobicity and have been both previously administered by starPEG‐HEP hydrogels.^[^ ^33^ ^]^ VEGF165 contains 42% of uncharged amino acids (A, C, F, I, L, M, P, V, W, and Y) and 53% of amino acids with polar side chains (D, E, H, K, N, Q, R, S, and T; 15% acidic and 19% basic of total amino acid residues). The aliphatic index (AI, defined as the molar ratio of aliphatic amino acids, dependent on the relative volume of the aliphatic side chain)^[^ ^34^ ^]^ of VEGF165 was determined to 50.2; the isoelectric point (i.e.p.) is 8.3. PDGF‐BB contains 50% uncharged amino acids (A, C, F, I, L, M, P, V, W, and Y) and 48% amino acids with polar side chains (D, E, H, K, N, Q, R, S, and T; 9% acidic and 18% basic) and is thus more hydrophobic than VEGF165. The AI for PDGF‐BB was determined to be 90.3, the isoelectric point (i.e.p.) is 9.4.

**Figure 4:**
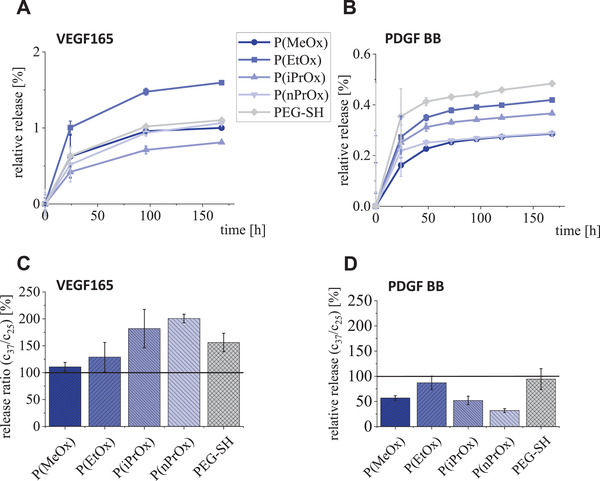
Growth factor release from POx‐HEP and PEG‐HEP hydrogels. A) Percentage release of VEGF165 at room temperature. B) Percentage release of PDGF‐BB at room temperature. C) Ratio of VEGF165 release at 37 °C to release at room temperature in percent. D) Ratio of PDGF‐BB release at 37 °C to release at room temperature in percent.

Incorporating the GFs upon gel formation in situ resulted in their quantitative uptake and homogenous distribution in either type of the POx‐/PEG‐HEP hydrogels, followed by the release of a minor (2%) fraction of both deployed growth factors (**Figure** **4**A,B). The overall low release of the GFs is in line with the huge excess of heparin binding sites (more than 500:1 assuming only one binding site per heparin) allowing for multiple binding/release/re‐binding steps. Decrease of the number of binding sites of heparin by partial desulfation has shown to enhance the release rate in comparable starPEG‐HEP hydrogels.^[^ ^6^ ^]^ The released amounts of VEGF165 were found to be significantly higher than of PDGF‐BB, which can be explained by the more basic characteristics of PDGF‐BB. The basic character results in a higher affinity to the GAG heparin as it was found for soluble heparin using micro‐thermophoresis or biolayer interferometry to determine the dissociation constants: *K*_d_ values of 40 × 10^−9^–165 × 10^−9^ m for VEGF165^[^ ^35^ ^]^ and 41 × 10^−9^ m for PDGF‐BB^[^ ^36^ ^]^ have been determined.

The cumulative VEGF‐release over 168 h from P(EtOx)‐HEP was significantly higher than P(MeOx) (*p*  =  0.042) and P(iPrOx)‐HEP (*p*  = 0.014), while nonsignificant differences have been found for the other hydrogel types (see Figure 4A). The P(EtOx) might shield heparin binding sites a bit more efficiently and thus prevent slightly more re‐binding of VEGF and thus result in slightly higher release. P(iPrOx) instead is more hydrophobic than P(EtOx) and leads to stronger unspecific hydrophobic interactions with proteins. It can be hypothesized that the modulation of network hydrophilicity via the choice of the synthetic polymer can be employed to control the release kinetics of the VEGF. For P(nPrOx)‐HEP, this trend was not observed, an effect that might be attributed to network defects as discussed above. For PDGF‐BB, the POx hydrophobicity correlated inversely with the released amounts of growth factor, and PEG‐HEP hydrogels showed the highest release (*p* = 0.002 vs P(nPrOx)), in accordance with the hydrophobicity ranking (Figure 4B).^[^ ^37^ ^]^ The most hydrophilic P(MeOx) showing the lowest release did not follow this trend, presumably due to steric restrictions, resulting from the shorter and thus more rigid polymer connections.

The release of VEGF165 was found to be enhanced by about 200% for the thermoresponsive hydrogels (P(iPrOx)‐HEP and P(nPrOx)‐HEP) when the temperature was elevated to 37 °C (Figure 4C). This effect is considered to be the consequence of a sponge‐like squeezing out of the thermoresponsive hydrogels as it has been discussed for temperature‐responsive hydrogels before.^[^ ^38^ ^]^ The higher release from PEG‐HEP hydrogels at 37 °C is attributed to a slightly enhanced swelling resulting from network defect structures since a lower solid content of the precursor mixture was used for gel formation and the heparin concentration was adjusted before hydrogel swelling at increased temperature.

In contrast, the release of PDGF‐BB was found to be reduced for the thermoresponsive hydrogels upon elevation of the temperature to 37 °C (Figure 4D). Thus, the increased hydrophobicity of the collapsed POx results in enhanced retention of the significantly more hydrophobic growth factor PDGF‐BB.

We conclude that thermoresponsive POx‐HEP gels allow for the temperature‐triggered regulation of growth factor release, however, in patterns that depend on the particular physicochemical characteristics of the protein and the resulting interactions with the polymer network. Together with the previously recorded uptake and release characteristics of GAG‐PEG hydrogels containing different concentrations of GAGs of different sulfation,^[^ ^3b^ ^]^ respectively, this finding creates unprecedented options for an even more precise growth factor management by means of affine biohybrid materials, as, e.g., applicable in the combinatorial customization of cell‐instructive matrices.

### 2.5 Bioadhesion to POx‐HEP Hydrogels

The adsorption of adhesion‐mediating proteins and the subsequent adhesion of cells and bacteria are basic features of any biomaterial to be considered for almost all potential applications. We, therefore, tested to what extent the variation of the POx component influenced the bioadhesive characteristics of POx‐HEP hydrogels.

**Figure 5:**
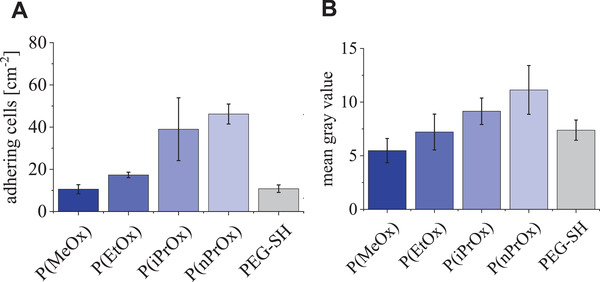
Fibroblast culture and fibronectin adsorption on POx‐HEP hydrogels and PEG‐HEP hydrogels at 37 °C. A) Fibroblast (L929) adhesion after 5 h of culture on POx‐HEP and PEG‐HEP layers (cell count based on light microscopy). B) Adsorption of TAMRA‐labeled fibronectin on POx‐HEP and PEG‐HEP layers (quantification based on mean gray value).

Adhesion experiments with L929 fibroblasts onto POx‐HEP hydrogel surfaces at 37 °C displayed higher cell numbers on the thermoresponsive hydrogels (P(iPrOx)‐HEP and P(nPrOx)‐HEP after 5 h incubation (**Figure** **5**A)). This effect can be attributed to the more hydrophobic characteristics of the collapsed hydrogels, which better anchor adhesion receptor ligand‐containing proteins adsorbed from the culture medium or from cell‐secreted matrices due to unspecific hydrophobic interactions. The experimentally observed enhanced adsorption of plasma fibronectin to the thermoresponsive hydrogels (P(iPrOx)‐HEP and P(nPrOx)‐HEP) confirmed this assumption (Figure 5B). The gradation of the adsorbed amounts of fibronectin qualitatively agrees with data reported by Zhang et al.^[^ ^13d^ ^]^ who studied grafted layers of the compared POx types. Thus, the collapsed thermoresponsive POx‐HEP hydrogels can effectively support cell adhesion and growth at physiological conditions due to their switchable hydrophilicity/hydrophobicity. The viability of the adherent cells on all tested gels was >80%, according to live/dead staining after 72 h of cell adhesion (Figure S7, Supporting Information).

**Figure 6:**
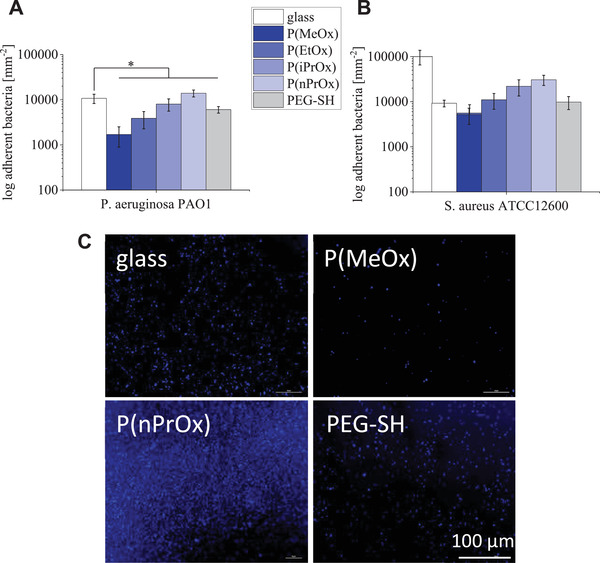
Bacteria adhesion on POx‐HEP and PEG‐HEP hydrogels at 37 °C. A) Average number of adhering *P. aeruginosa* (strain PAO1) on POx‐HEP and PEG‐HEP layers ((10^4^ µm^2^)^−1^). B) Average number of adhering *S. aureus* (strain ATCC12600) on POx‐HEP and PEG‐HEP layers ((10^4^ µm^2^)^−1^). C) Representative fluorescent microscopy images of DAPI‐stained nucleic acid for *S. aureus* (strain ATCC12600) on glass, P(MeOx)‐, P(nPrOx)‐, and PEG‐HEP layers.

POx coatings were previously reported to be beneficial for minimizing bacterial adhesion to degrees even exceeding the effect of PEG coatings.^[^ ^13^, ^39^ ^]^ To explore the related characteristics of POx‐HEP and PEG‐HEP layers, we tested the adhesion of *E. coli* and *P. aeruginosa* (gram‐negative) as well as *S. aureus* and *S. epidermidis* (gram‐positive bacteria) at physiological temperature of 37 °C. Glass, on which the hydrogels were coated, was used as control. All hydrogels, except P(nPrOx)‐HEP, showed a lower adhesion of *P. aeruginosa*, a strong gram‐negative biofilm‐forming bacterial strain, compared to glass (**Figure** **6**A). For *S. aureus*, a very strong gram‐positive biofilm former, only P(MeOx)‐HEP caused a lower adhesion when coated on glass (Figure 6B). Fluorescence microscopy images of 4′,6‐diamidino‐2‐phenylindole (DAPI) stained nucleic acid for *S. aureus* (Figure 6C) revealed a nearly complete coverage of P(nPrOx)‐HEP and only few visible stains on P(MeOx)‐HEP. The bacterial cell counts for *E. coli* and *S. epidermidis* on the gel surfaces are given in Figure S8 in the Supporting Information; these less biofilm‐forming bacterial strains only minimally attached to the POx‐HEP materials, however, in a comparable ranking as it has been found for the strong biofilm formers.

In general, increasing hydrophobicity of the POx component resulted in increased bacterial settlement onto the respective POx‐HEP gels. The bacterial adhesion to PEG‐HEP hydrogels was found to range in between the adhesion to P(EtOx)‐ and P(iPrOx)‐HEP hydrogels. Hydrophilic POx‐HEP coatings can in fact reduce bacterial adhesion, however, to degrees that strongly depend on the particular bacterial strain.

### 2.6 Hemocompatibility of POx‐HEP Hydrogels

To evaluate the suitability of POx‐HEP materials for blood‐contacting applications, we tested the response of human whole blood to the in vitro incubation of the hydrogels. Blood activation parameters after 2 h of incubation at 37 °C were compared with different reference surfaces well‐known for their low activation potential (Teflon AF and PEG‐HEP hydrogels).^[^ ^8b^ ^]^

None of the materials induced hemolysis, as the blood plasma after centrifugation was obtained as clear yellowish fluid.

**Figure 7:**
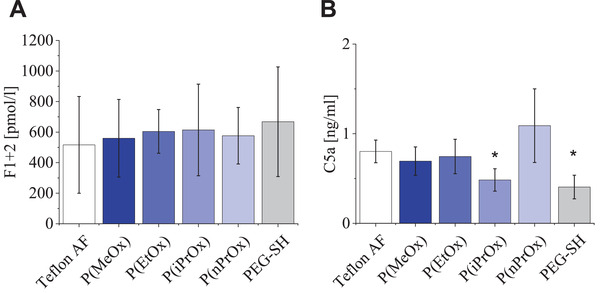
Hemocompatibility of POx‐HEP and PEG‐HEP materials as determined by human whole blood incubation in vitro. A) F1+2 concentration after incubation. B) C5a concentration after incubation. * indicates significant difference (*p* < 0.05) against P(nPrOx), P(EtOx), and Teflon AF in ANOVA on ranks (*n*  =  9).

To test for hemostasis, we compared the POx‐HEP materials with respect to plasmatic coagulation activation. During the activation of prothrombin to thrombin, a key step of the coagulation system, fragment F1+2 is cleaved off^[^ ^40^ ^]^ and can thus be used as a soluble marker of thrombin activation. All POx‐/PEG‐HEP hydrogels caused comparable levels of F1+2 as the Teflon AF reference surfaces, indicating low levels of coagulation activation. There were no significant differences between the compared POx‐HEP materials at the applied experimental conditions (**Figure** **7**A), pointing at the good accessibility of the heparin at the hydrogel surfaces, independent of the thermoresponsive swelling of the gel.

Thrombin is among the strongest activators of blood platelets. The number of conjugates formed from activated platelets with granulocytes as an indicator of platelet activation, a process that is known to amplify the coagulation system, was determined by flow cytometry. PEG‐HEP hydrogels and Teflon AF coatings caused the formation of a similar number of conjugates. All utilized POx‐HEP hydrogels resulted in significantly lower numbers of blood platelet conjugates with granulocytes and monocytes compared to the PEG‐HEP and Teflon AF reference surfaces (25% aggregates formed for all POx‐HEP gels surfaces, 50% aggregates formed for PEG‐HEP gel surface and Teflon AF; percentage of platelet‐leukocyte conjugates shown in Figure S9A, Supporting Information). Finally, platelet activation was determined via the secretion of the soluble platelet factor 4 (PF4, CXCL4).^[^ ^41^ ^]^ For all POx‐HEP hydrogels, significantly lower levels of PF4 in comparison to Teflon AF were detected, confirming lower platelet activation for these hydrogel‐coated surfaces (results shown in Figure S9B, Supporting Information).

Biomaterials exposed to blood can activate the innate immune system, which consists of complement proteins and leukocytes.^[^ ^42^ ^]^ Activation of the complement system as the plasmatic part of the innate immune response leads to the cleavage of the C5 molecule into a surface‐bound C5b and soluble C5a, the latter attracting and activating monocytes and neutrophils.^[^ ^43^ ^]^ The C5a concentration in blood after exposure to hydrogels was the lowest for PEG‐ and P(iPrOx)‐HEP gels, followed by P(MeOx)‐, P(EtOx)‐HEP materials with concentrations around 0.6–0.7 ng mL^−1^ C5a. Over all tested hydrogels, P(nPrOx)‐HEP induced the highest concentration of C5a with 1.5 ng mL^−1^ (Figure 7B). As a second parameter for inflammation, we tested the expression of CD11b, which, as a neutrophil surface antigen, is quickly upregulated when endotoxins, bacteria, or activated complement are present and thus is widely used as a marker for leukocyte activation.^[^ ^44^ ^]^ For the applied hydrogels, the expression of this activation marker was lowest for P(MeOx)‐ and P(iPrOx)‐HEP, followed by P(EtOx)‐, P(nPrOx)‐, and PEG‐HEP hydrogels. All samples caused significantly lower leukocyte activation than Teflon AF surfaces (Figure S9C, Supporting Information). The decay of granulocyte numbers as a marker of the adhesion of activated immune cells confirmed the above CD11b data: The lowest granulocyte loss was determined for POx‐HEP gels (about 12%) without significant differences between the compared POx‐HEP material variants. PEG‐HEP hydrogels showed a slightly higher decrease, comparable to the effect of Teflon AF‐coated surfaces (about 20% decay; Figure S9D, Supporting Information).

In sum, all POx‐HEP hydrogels offer excellent hemocompatibility concerning blood coagulation and inflammatory response, clearly outperforming the benchmark coating material Teflon AF. This superior antithrombotic and anti‐inflammatory property is independent of the thermoresponsiveness and the hydrophobicity of the gels and can be attributed to the high concentration of heparin at the hydrogel surface. The anticoagulant action of heparin results from the complexation with antithrombin, and the anti‐inflammatory action is a consequence of the binding of complement factor H. Antithrombin has been shown to bind mainly to the surface of heparin‐containing hydrogels with minor diffusion to the bulk due to its high molecular weight and the specific affinity to the pentasaccharide sequence of heparin. Factor H is a large molecule with 155 kDa molecular weight limiting the penetration into hydrogels of the mesh size investigated in this study. The swelling state of the POx‐HEP hydrogels, therefore, does not influence its hemocompatibility.

When compared to PEG‐HEP materials, POx‐HEP gels performed similar or distinctly better (platelet activation as measured by PF4 release). It is thus very attractive to expand the current study and, e.g., use the introduced POx‐HEP gels as coagulation enzyme‐cleavable autoregulative heparin release system along the lines of our recently explored adaptive PEG‐HEP systems,^[^ ^8c^ ^]^ which were shown to afford anticoagulant functionality regulated by the actual demand.

## 3 Conclusion

POx‐HEP hydrogels were produced by a cytocompatible Michael‐type addition from POx of gradated characteristics, offering a far‐reaching tunability of the obtained materials’ properties. In particular, the temperature‐switchable polymer network structure and the local hydrophobicity were demonstrated to be adjustable independent of the concentration of the strongly charged, bioactive compound HEP. This provides selectivity of the temperature‐triggered release of growth factors from the temperature‐responsive network depending on their physicochemical characteristics, as the more hydrophobic and basic PDGF‐BB was entrapped, whereas VEGF165 was squeezed out at elevated temperature. The temperature‐responsiveness of the POx component in the hydrogel can impart this biohybrid hydrogel with further specific features that are not analyzed here. POx‐HEP hydrogels expand the options of previously developed biohybrid starPEG‐HEP hydrogels and are considered an attractive alternative to PEG‐based hydrogels. Further dedicated studies can therefore rely on the advantages of the physicochemical variability of POx in the design of specifically customized biohybrid hydrogel materials containing sulfated glycosaminoglycans.

## 4 Experimental Section

### Materials and Methods

All chemicals were purchased from Sigma‐Aldrich (Weinheim, Germany) and used as received unless otherwise stated. Acetonitrile (ACN), triethylamine (TEA), and the 2‐alkyl‐2‐oxazolines for polymerization were used after reflux over CaH_2_ and distilled under nitrogen. The initiator *trans*‐1,4‐dibromobut‐2‐ene (DBB) was used after recrystallization from dry acetone and drying in vacuum.

Gel permeation chromatography (GPC) was performed as described previously.^[^ ^45^ ^]^ Briefly, sample separation was performed with a precolumn (GRAM, 8 mm x 50 mm, 10µ) and two GRAM 1000 columns (8 mm x 300 mm 10µ)) (PSS, Mainz, Germany). Chromatographic analysis was on a PL‐GPC 120 column with an eluent of dimethyl acetamide (DMAc) containing 5 g L^−1^ LiBr and 1% Milli‐Q water (Merck, Darmstadt, Germany) at a flow rate of 1 mL min^−1^ at 70 °C. A volume of 100 µL was injected and a refractive index (RI) detector was used for analysis. PMeOx standards were used for calibration in the range of 949–16 500 g mol^−1^ (Table S5.1, Supporting Information). (P(MeOx)_9_ (949 g mol^−1^), P(MeOx)_19_ (1800 g mol^−1^), P(MeOx)_27_ (2482 g mol^−1^), P(MeOx)_50_ (4536 g mol^−1^), P(MeOx)_93_ (8119 g mol^−1^), P(MeOx)_200_ (16 500 g mol^−1^)).

Matrix‐assisted laser desorption ionization time‐of‐flight mass spectrometry (MALDI‐ToF‐MS) was performed on a Biflex IV from Bruker Daltonics (Billerica, USA). 1 mg mL^−1^ analyte in methanol containing 0.1 vol% trifluoroacetic acid was mixed with a saturated solution of sinapinic acid in methanol in a ratio of 1:5 and 1 µL and placed on the target. The system was calibrated against the peptide calibration standard II (Bruker) at sample conditions.

^1^H NMR spectroscopy was performed on a Bruker DRX 500 P at 500 MHz and 298 K, using D_2_O, CDCl_3_, and DMSO‐d_6_ as solvents. The spectra were calibrated to the tetramethylsilane signal.

Differential scanning calorimetry (DSC) was carried out on a Micro DSC III (Setaram, Caluire‐et‐Cuire, France) using two stainless steel microcapsules for sample and reference. For polymer samples in solution, the analyte was dissolved in a concentration of 1 mg mL^−1^ in 750 µL phosphate‐buffered saline (PBS). For calorimetric measurements of the hydrogels, the concentration of the synthetic polymer inside the capsule was adjusted to 1 mg mL^−1^, and accordingly, the volume of the gel and of additional PBS was determined. 750 µL PBS was used as a reference. Measurements were carried out in between 10 and 60 °C.

AFM measurements were performed as described previously,^[^ ^46^ ^]^ using a Nanowizard II AFM (JPK Instruments, Berlin, Germany) mounted on an inverted optical microscope (Observer.D1, Zeiss, Jena, Germany) at room temperature (25 °C) or 37 °C using a PetriDishHeater (JPK Instruments, Berlin, Germany). Measurements were performed with tipless silicon nitride cantilevers with a nominal spring constant of 80 mN m^−1^ (PNP‐TR‐TL‐Au; Nanoworld, Neuenburg, Switzerland), modified with silica beads (∅10 µm, Kisker Biotec GmbH, Steinfurt, Germany) after calibration of the spring constants.^[^ ^47^ ^]^ A closed‐loop, constant height mode with 3 nN contact force and 5 µm s^−1^ approach/retract velocity was used to record the force–distance curves. A minimum of 45 spots per sample was recorded. The software of the AFM manufacturer (JPK Instruments) was used to calculate the elastic modulus E from the approach force–distance curves.

Rheological measurements were performed on a rotational rheometer from TA Instruments (Eschborn, Germany) applied with an 8 mm parallel plate geometry. The disks turned in a frequency of 10^−1^ to 10^−2^ rad s^−1^ and a strain of 2%. The initial force applied to the sample was adjusted to minimal slipping effects. The internal calculation of the instrument software was used to acquire the storage modulus *G’*.

UV/VIS measurements were performed on an UV‐DU800 Spectrophotometer (Beckman‐Coulter, Brea, USA) at room temperature, measuring the absorption of Ellman's reagent at 412 nm in PMMA cuvettes (pathlength 1 cm) for determining the thiol group reactivity as described in the protocol derived from Thermo Fisher. For concentration calculation, the extinction coefficient of 5‐thio‐(2‐nitrobenzoic acid) in 0.1 m sodium phosphate, pH 8.0, containing 1 × 10^−3^ m ethylenediaminetetraacetic acid (14 150 M^−1^ cm^−1^) was inserted to Beer–Lambert law.

Fluorescence scanning images were recorded to determine the hydrogel diameter using a FLA 5100 (Fujifilm, Tokyo, Japan) with excitation at 473 nm and emission filter of 510 nm long pass. The diameter was obtained using the program MultiGauge.

### Synthesis of Poly(2‐alkyl‐2‐oxazolines)

The polymerization reaction was carried out under dry and inert conditions, using Schlenk and glovebox techniques for preventing undesired termination. Hence a mixture of 1 eq DBB as initiator and the desired monomer amount of 2‐alkyl‐2‐oxazoline (2‐methyl‐, 2‐ethyl‐, 2‐iso‐propyl‐ and 2‐*n*‐propyloxazoline) in AcN were heated in a Schlenk flask to 80 °C in a silicon oil bath for an adequate time period, afterward the mixture was cooled in an ice bath, and the termination reagent was added. The degree of polymerization (DP*_n_*) was calculated by the ratio of 2‐alkyl‐2‐oxazoline monomer [M_0_] to initiator [I_0_]. The initial monomer concentration is adjusted to 3 m. For termination, potassium thioacetate (3 eq regarding DBB) and triethylamine (TEA; 4 eq regarding DBB) were added to the polymerization mixture and allowed to react overnight at ambient temperature. For purification, AcN was removed under reduced pressure, and the polymer was dialyzed against MilliQ (filtrated deionized water) to remove residues of the terminating agents. Subsequent to lyophilization, white to slightly yellowish polymer powders were obtained.

^1^H NMR spectra are presented in Figures S1 and S2 in the Supporting Information

Poly(2‐methyl‐2‐oxazoline)—P(MeOx)*_n_*: ^1^H NMR (CDCl_3_), 400 MHz, *δ* [ppm]: 5.67–5.44 (m, 2 H), 4.04–3.84 (m, 4 H), 3.83–3.21 (m, n⋅4 H), 3.06–2.72 (m, 4 H), 2.46–2.30 (m, 6 H), 2.23–1.84 (m, n⋅3 H).

Poly(2‐ethyl‐2‐oxazoline)—P(EtOx)*_n_*: ^1^H NMR (CDCl_3_), 400 MHz, *δ* [ppm]: 5.62–5.42 (m, 2 H), 4.05–3.79 (m, 4 H), 3.76–3.07 (m, n⋅4 H), 3.05–2.84 (m, 4 H), 2.49–2.18 (m, n⋅2 H + 6 H), 1.18–0.96 (m, n⋅3 H).

Poly(2‐n‐propyl‐2‐oxazoline)—P(nPrOx)*_n_*: ^1^H NMR (CDCl_3_), 400 MHz, *δ *[ppm]: 5.62–5.39 (m, 2 H), 4.03–3.81 (m, 4 H), 3.73–3.07 (m, n⋅4 H), 3.05–2.85 (m, 4 H), 2.42–2.13 (m, n⋅2 H + 6 H), 1.72–1.49 (m, n⋅2 H), 1.03–0.78 (m, n⋅3 H).

Poly(2‐iso‐propyl‐2‐oxazoline)—P(iPrOx)*_n_*: ^1^H NMR (CDCl_3_), 400 MHz, *δ* [ppm]: 5.64–5.40 (m, 2 H), 4.06–3.80 (m, 4 H), 3.70–3.07 (m, n⋅4 H), 3.05–2.47 (m, n⋅1 H + 4 H), 2.40–2.24 (m, 6 H), 1.20–0.69 (m, n⋅6 H).

The molecular weight of the polymers was calculated as followed

(1) $$\begin{array}{ccc} n & = & \frac{\mathrm{peak}\;\mathrm{area}\;\mathrm{of}\;\mathrm{repeating}\;\mathrm{polymer}\;\mathrm{sidechain}\left(c,d,e,\left(f,g\right)\right)}{\mathrm{peak}\;\mathrm{area}\;\mathrm{of}\;\mathrm{protons}\;\mathrm{on}\;\mathrm{double}\;\mathrm{bond}\;\mathrm{of}\;\mathrm{initiator}\;\left(a,1H\right)}\\ & & *\frac{\mathrm{number}\;\mathrm{of}\;\mathrm{protons}\;\mathrm{on}\;\mathrm{double}\;\mathrm{bond}\;\mathrm{of}\;\mathrm{initiator}\left(a,\;1H\right)}{\mathrm{number}\;\mathrm{of}\;\mathrm{protons}\;\mathrm{on}\;\mathrm{sidechain}\left(c,d,e,\left(f,g\right)\right)} \end{array}$$

(2) $$\begin{array}{c} M_{n}=2*MW_{\mathrm{termination}\;\mathrm{group}}+MW_{\mathrm{encapsulated}\;\mathrm{inititator}}+n*MW_{\mathrm{repeting}\;\mathrm{unit}} \end{array}$$

In this formula *n* is the number of repetition units, which can be calculated by multiplying the ratio of the area under the signals for the respective POx repetition units (c, d, e, (f and g)) to the area under the signal for the double bond (a; between 5.6 and 5.4 ppm) with the ratio of the number of protons at the double bond (signal a) to the number of protons at the respective POx repetition unit (c, d, e, (f and g)). To obtain the molecular weight, twice the molecular mass of the termination group (thioacetate, 75 g mol^−1^), was added to the molecular mass of the encapsulated initiator (but‐2‐enyl, 54 g mol^−1^) and added to *n*‐times the molecular weight of the repeating unit (MeOx, 89 g mol^−1^; EtOx, 103 g mol^−1^; iPrOx, 118 g mol^−1^; nPrOx, 117 g mol^−1^).

The endgroup fidelity of the polymers was calculated as followed

(3) $$\begin{array}{ccc} & & \mathrm{Endgroup}\;\mathrm{fidelity}=\\ & & \frac{\mathrm{peak}\;\mathrm{area}\;\mathrm{of}\;\mathrm{protons}\;\mathrm{on}\;\mathrm{methyl}\;\mathrm{group}\;\mathrm{of}\;\mathrm{thioacetate}}{\mathrm{number}\;\mathrm{of}\;\mathrm{protons}\;\mathrm{on}\;\mathrm{methyl}\;\mathrm{group}\;\mathrm{of}\;\mathrm{thioacetate}\left(3H\right)}/\\ & & \frac{\mathrm{peak}\;\mathrm{area}\;\mathrm{of}\;\mathrm{protons}\;\mathrm{on}\;\mathrm{double}\;\mathrm{bond}\;\mathrm{of}\;\mathrm{initiator}\left(a,\;1H\right)}{\mathrm{number}\;\mathrm{of}\;\mathrm{protons}\;\mathrm{on}\;\mathrm{double}\;\mathrm{bond}\;\mathrm{of}\;\mathrm{initiator}\left(a,\;1H\right)}*100 \end{array}$$

To determine the percentage of turned over endgroups, the ratio of the signal peak area on the methyl group of the thioacetate to the number of protons on this methyl group is divided by the ratio of the peak area of the protons on the double bond of the initiator (signal a) to the number of protons on the initiator (signal a). By multiplying with 100 percentage was obtained.

*Removal of Acetyl Protection Group for Obtaining Dithiol‐Terminated Poly(2‐alkyl‐2‐oxazolines)*: The hydrolysis of thioesters could be achieved under basic or acidic conditions. The acetylated lyophilized polymer samples were dissolved in ice‐cooled MilliQ water or methanol, from which the required solvent volume was calculated for a total hydrolysis reagent concentration of 2 m. As hydrolyzing agents NaOH, KOH, NH_4_OH, NaHCO_3_, trifluoroacetic acid (TFA), acetyl chloride (AcCl), and HCl were chosen, which were added in a 80 times excess to polymer to the cooled solution, brought to ambient temperature and stirred for 1 h. For preventing undesired side reactions, a vigorous N_2_ flow was applied, leading to the removal of volatile compounds. Free thiol groups tended to undergo air oxidation when presented as deprotonated ion under basic condition, resulting in disulfide bonds or even higher oxidized sulfur moieties. In order to prevent side reactions, the raw product was dissolved in MilliQ water and mixed with five times excess of TCEP to polymer, subsequently pH was adjusted to 7 and stirred for 1 h. 2 m NaCl was added; afterward the reaction had to be dialyzed once against deionized water, next three times against 1 m NaCl and finally five times against deionized water. Additionally, the dialysis solution was bubbled with N_2_ for preventing oxidation. Subsequent to lyophilization, white powderous polymers were obtained.

*Synthesis of Heparin‐Maleimide*: The synthesis of heparin‐maleimide was carried out according to Tsurkan et al.^[^ ^7^, ^36^ ^]^ Briefly, 1.5‐fold excess of s‐NHS (*N*‐hydroxysulfosuccinimide) and threefold excess of EDC to amount of maleimide groups potentially conjugated were added to heparin (HEP, Sodium salt, Porcine Intestinal Mucosa, Merck, Darmstadt, Germany) dissolved in MilliQ. The activation was carried out for 20 min at 5 °C. Subsequently, the desired amount of *N*‐(2‐aminoethyl)maleimide trifluoroacetate salt was dissolved in MilliQ, added to the activated heparin, and stirred overnight. The heparin‐maleimide was purified by dialysis three times against 1 m sodium chloride and three times against MilliQ. The product was obtained by lyophilization as a slightly yellow powder. The conjugation degree was verified by ^1^H NMR as described before.^[^ ^36^ ^]^

*Hydrogels*: Hydrogels were formed from linear PEG dithiol‐ (PEG‐SH, MW: 2500 g mol^−1^, JenKem Technology, Plano, USA) or the above‐described POX dithiol‐polymers and HEP‐maleimide by Michael‐type addition in a protocol adapted from Tsurkan et al.^[^ ^7^ ^]^ PEG/POx dithiol and HEP‐maleimide were dissolved in ice‐cooled PBS, adjusting the concentration to equimolar content of thiol groups to maleimide groups considering that the solid content of the hydrogels was restricting the volume of the gel solution. Hence for keeping the HEP‐maleimide concentration constant, the solid content had to be adjusted. Different from the previously reported protocol for the starPEG‐HEP gel formation,^[^ ^24a^ ^]^ an excess of thiol‐terminated polymer was added to overcome the lack of thiol reactivity, however, this excess was not involved in the solid content calculation. After full solvation of both polymers, equal volumes were mixed, and gelation took place within 5 min. To complete the reaction, the gels were kept for another 30 min, subsequently immersed, and swollen overnight in PBS. By determining the swollen diameter of the gel using fluorescence scanning imaging, the swelling degree *Q* was calculated by using *Q  =  V*/*V*_0_  = (*d/d*_reac_)^3^ *V*_reac_/*V*_0_, with *d* as diameter of the swollen gel disk, *d*_reac_ as diameter of the unswollen gel disk (cured reaction mixture), *V*_reac_ as volume of the cured reaction mixture, and *V*_0_  =  *nν*_PEG_ + *nν*_HEP_ is the volume of the dry gel.

### Equilibrium Shrinking of Thermoresponsive P(iPrOx) and P(nPrOx)

After equilibrium swelling, the hydrogel disks were stored in PBS at 37 °C overnight for equilibrium shrinking at elevated temperature. Subsequently, the hydrogel diameter was determined by a light microscopy stage (S8APO connected to DFC295 camera, Leica, Wetzlar, Germany) equipped with a piezo heating element at 37 °C (Ostech, Berlin, Germany). The diameter was calculated using ImageJ calibrated with scale paper.

### Growth Factor Loading and Release

HEP‐maleimide and POx/PEG‐thiol solutions were prepared as described above for the hydrogel synthesis, whereas the heparin concentration was kept constant over all parameters. Recombinant human vascular endothelial growth factor 165 (VEGF) and recombinant human platelet‐derived growth factor‐BB (PDGF) (both obtained from Peprotech, Rocky Hill, USA) were diluted in PBS to a concentration of 1 µg µL^−1^. Subsequently, heparin and growth factor solution were combined, resulting in 1:500 molar ratio of growth factor to heparin. With this mixture, hydrogels were formed by combining both hydrogel components in protein low binding tubes (Eppendorf, Wesseling‐Berzdorf, Germany), resulting in 10 µL total gel volume (*n* = 3). After full gelation, the gels were rinsed with Dulbecco's modified Eagle medium (DMEM) high glucose cell culture medium containing 0.1% bovine serum albumin (BSA) and Proclin 300 (release medium) twice and finally stored in 300 µL release medium for growth factor release at room temperature and 37 °C, respectively. The release profile was determined by measuring the growth factor concentration in the supernatant, which was completely removed every 24 h, immediately frozen at −80 °C, and replaced by 300 µL fresh release medium. Each sample was assayed in duplicate using an enzyme‐linked immunosorbent assay (ELISA) Quantikine kit (R&D Systems, Minneapolis, USA).

### Protein Adsorption and Cell Adhesion

Fibronectin was labeled with FluoReporter 5‐carboxytetramethylrhodamine (5‐TAMRA) labeling kit (Molecular Probes), according to the supplier protocol, and after purification diluted to 50 µg mL^−1^ in PBS. Equilibrium swollen hydrogel disks (1.3 cm^2^ per gel; *n* = 3) were immersed for 1 h in the diluted protein solution. After rinsing twice with PBS, samples were analyzed using fluorescence microscopy.

*Fluorescence Microscopy*: The amount of adsorbed protein was analyzed by placing the gel surfaces reversed on microscopy slides using immersion oil. Imaging was carried out on Axio Observer Widefield microscope (Zeiss, Jena, Germany) using a 10X air objective. The samples were excited at 547 nm, and images were taken at an emission wavelength of 576 nm. The whole area was captured for each gel, and ImageJ was used to measure the mean gray value over the entire hydrogel surface.

*Cell Culture*: Cryo preserved fibroblasts (L929 or NIH/3T3) were acquired from American Type Culture Collection (ATCC). The cells were thawed and resuspended in cell culture medium as recommended. Culture was carried out at 100% humidity and 37 °C on standard T25 Tissue Culture Flasks for a maximum of about six to seven passages. The medium, for L929 RPMI 1640 (PAN Biotech, Aidenbach, Germany) and for NIH/3T3 DMEM high glucose (ThermoFisher Scientific, Waltham, USA) each additionally with 10% fetal bovine serum (Biochrom, Berlin, Germany) and 1% penicillin‐streptomycin, was exchanged every 2–3 days.

Hydrogel disks (1.3 cm^2^ per gel; *n* = 3) were swollen in PBS overnight, transferred to fibroblast growth medium, and incubated at 37 °C for 1 h. Fibroblasts (L929) were trypsinized, diluted to 5 × 10^3^ cells mL^−1^, and overlaid on the hydrogels. For determining the amount of initially adhering cells, light microscopic images were taken after 5 h.

### Bacterial Adhesion

Hydrogel disks (1.3 cm^2^ per gel; *n* = 3) were incubated in lysogeny broth (LB) medium at 37 °C overnight for equilibrium swelling. Bacterial strains of *E. coli, S. aureus, S. epidermidis*, and *P. aeruginosa* were grown in LB medium at 37 °C with shaking at 200 rpm overnight. The bacteria suspensions were centrifuged, washed, resuspended, and diluted to OD of 0.05. Next, the hydrogels were overlaid by bacteria suspension and incubated for 3 h at 37 °C. Subsequently, the growth solution was removed; the gels were rinsed four times with PBS, and the bacteria were fixed using 4% paraformaldehyde (PFA) in PBS for 15 min. After another rinsing step with PBS, 0.02 mg mL^−1^ DAPI was added, incubated for 20 min, the solution was carefully removed, and the samples were washed with PBS again for removal of unbound dye.

The stained gel disks were placed head down on microscopy slides using immersion oil. Imaging was carried out on Axio Observer Widefield microscope (Zeiss, Jena, Germany) using a 20X air objective. Excitation was achieved at 360 nm, and images were taken at an emission wavelength of 460 nm. For each gel, four different positions were captured, resulting in 12 pictures per condition. ImageJ was used to count the bacterial cells adhering on the gel surface.

### Hemocompatibility Assessment

The whole blood incubation was performed as described before.^[^ ^48^ ^]^ All studies were approved by the ethics board and complied with institutional and international guidelines (EK‐BR‐24/18‐1, Ethics committee of Sächsische Landesärztekammer, Dresden, Germany). Two independent incubations were performed with three parallel sets of samples, each. Blood was pooled from a pair of ABO‐matched volunteers, which was different between the experiments. The donors did not take any medication; especially, they had not taken nonsteroidal anti‐inflammatory drugs in the past 10 days.

Fully hydrated PEG/POx hydrogel (*n* = 6) disks were mounted to either side of a 6.4 mm thick incubation chamber, leaving 6.2 cm^2^ hydrogel surface for direct blood contact. The chambers were initially incubated in PBS at 37 °C until usage, resulting in equilibrium swollen gel disks, according to the individual temperature dependency. Subsequently, 2 mL of freshly drawn heparin‐anticoagulated (1.5 IU mL^−1^) whole blood was carefully injected into each chamber, avoiding air bubbles inside the chamber. The incubation was carried out at 37 °C for 2 h in constant overhead rotation with about six revolutions min^−1^. The blood samples were regained from the chamber, three replicates of each sample were mixed with the stabilizers recommended in the manuals of the ELISA test kits. After centrifugation, the plasma was stored at −80 °C until analysis. Plasma coagulation and blood platelet activation were determined by measuring the amount of prothrombin F1+2 fragment (Enzygnost F1+2 micro, Siemens, Eschborn, Germany) and platelet factor 4 (PF4, Zymutest PF4, CoaChrom, Vienna, Austria). Complement activation was determined by measuring the C5a fragment (DRG Instruments, Marburg, Germany) by ELISA. The cell surface markers CD11b (clone ICRF44, BioLegend, San Diego, California) and CD41a (clone HIP8, Becton Dickinson, Heidelberg, Germany) were analyzed by flow cytometry (LSR Fortessa Cell Analyzer, Becton Dickinson, Heidelberg, Germany) to determine leukocyte activation and granulocyte‐platelet conjugate‐formation, respectively. Detailed information for flow cytometric analysis was described previously.^[^ ^48b^ ^]^ The loss of blood cells during incubation was determined using a cell counter (Ac‐T diff., Beckman Coulter, Krefeld, Germany).

### Statistical Analysis

Data were analyzed using OriginLab 2018 (OriginLab Corporation, Northhampton, USA), multiple samples were evaluated by one‐way analysis of variance (ANOVA) followed by Tukey post hoc tests to evaluate the statistical differences (*p* < 0.05) among all samples or between samples and controls, respectively. All error bars given were standard deviations (SD).

## Conflict of Interest

The authors declare no conflict of interest.

## Supporting information

Supporting Information

## Data Availability

Research data are not shared.
