# Supplementary material for: Poly(2‐alkyl‐2‐oxazoline)‐Heparin Hydrogels—Expanding the Physicochemical Parameter Space of Biohybrid Materials
Source: Adv Healthc Mater. 2021 Sep 20;10(22):2101327. doi: 10.1002/adhm.202101327 (PMC11481032; doi:10.1002/adhm.202101327)
Supplement: Supplementary file 1 — Supporting Information [file ADHM-10-2101327-s001.pdf]

© 2021 Wiley-VCH GmbH

ADVANCED HEALTHCARE MATERIALS

Supporting Information

for *Adv. Healthcare Mater.,* DOI: 10.1002/adhm.202101327

Poly(2-alkyl-2-oxazoline)-heparin hydrogels – expanding the physicochemical parameter space of biohybrid materials

*Dominik Hahn, Jannick M. Sonntag, Steffen Lück, Manfred F. Maitz, Uwe Freudenberg, Rainer Jordan, Carsten Werner**

Supporting information

Poly(2-alkyl-2-oxazoline)-heparin hydrogels – expanding the physicochemical parameter space of biohybrid materials

*Dominik Hahn, Jannick M. Sonntag, Steffen Lück, Manfred F. Maitz, Uwe Freudenberg, Rainer Jordan, Carsten Werner**

Synthesis of Poly-(2-alkyl-2-oxazolines)

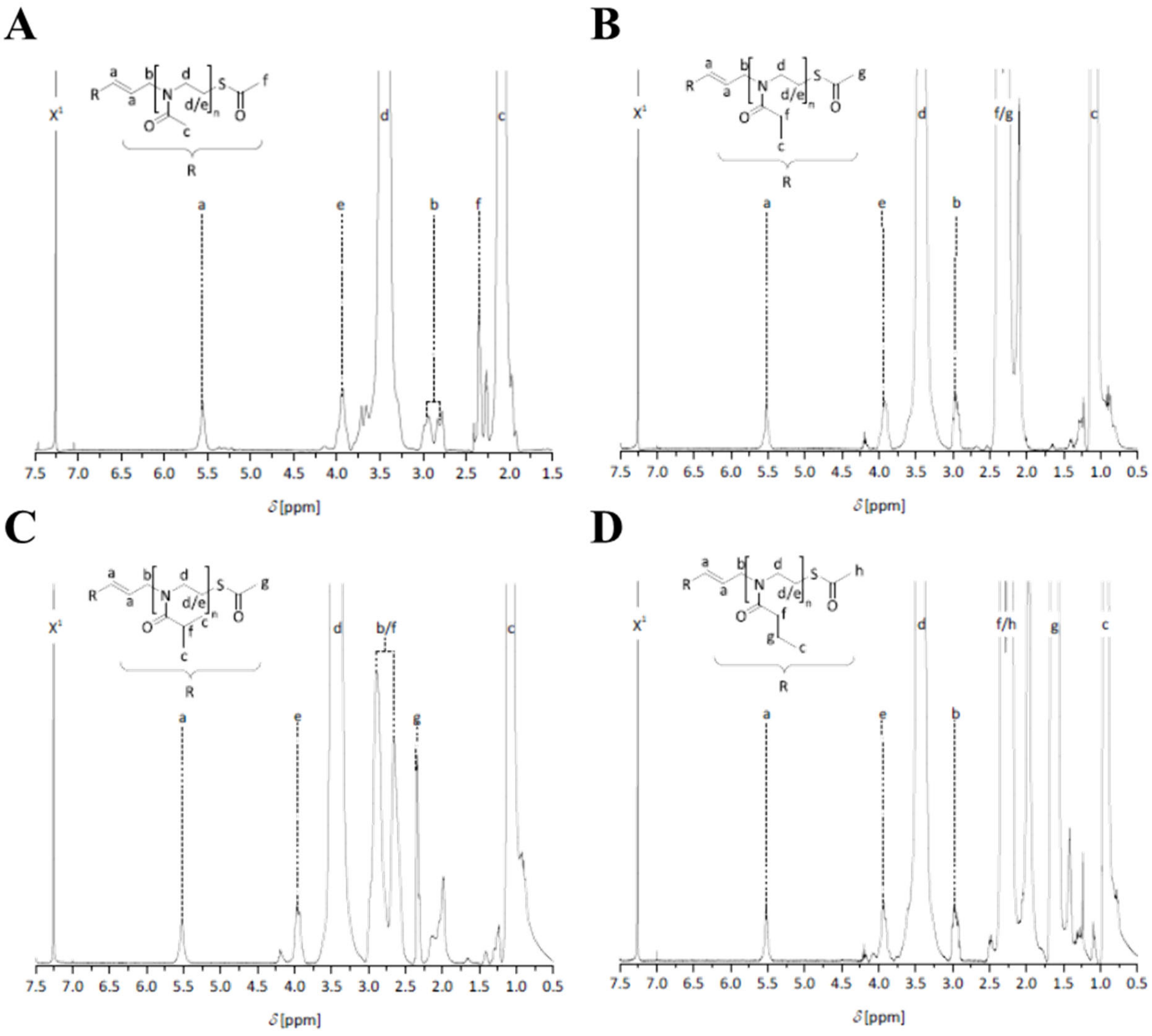

Supporting Figure 1: ^{1}H-NMR-spectra of thioacetate protected A: P(MeOx), B: P(EtOx)), C: P(iPrOx), D: P(nPrOx) dithiol; where the single letters determine the protons at the single position and X^1 is the solvent signal.

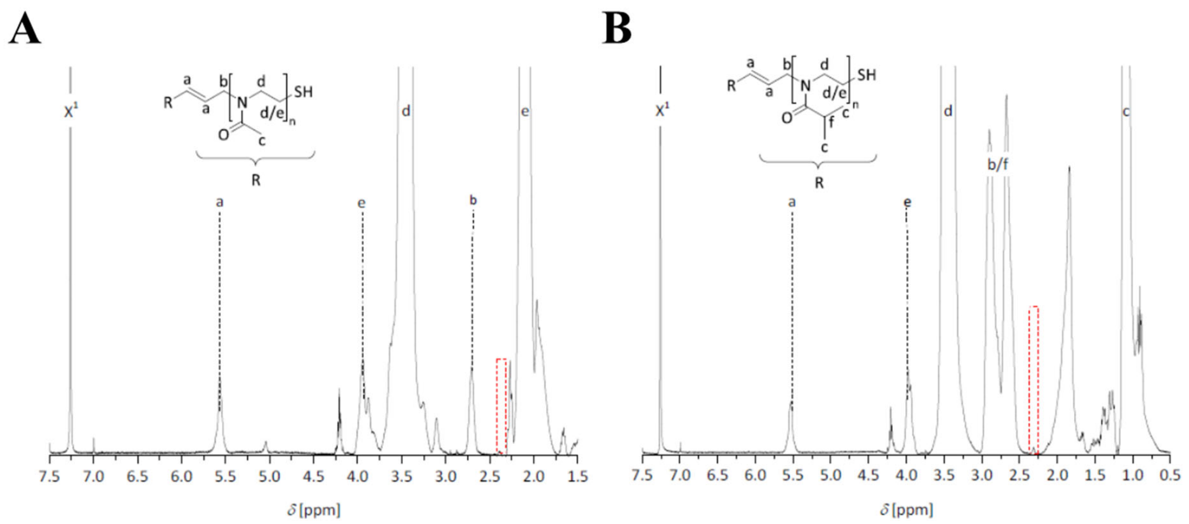

Supporting Figure 2: ^{1}H-NMR-spectra of deprotected A: P(MeOx), B: P(iPrOx) dithiol; where the single letters determine the protons at the single position, X^1 is the solvent signal and the red square locates the vanished protection group.

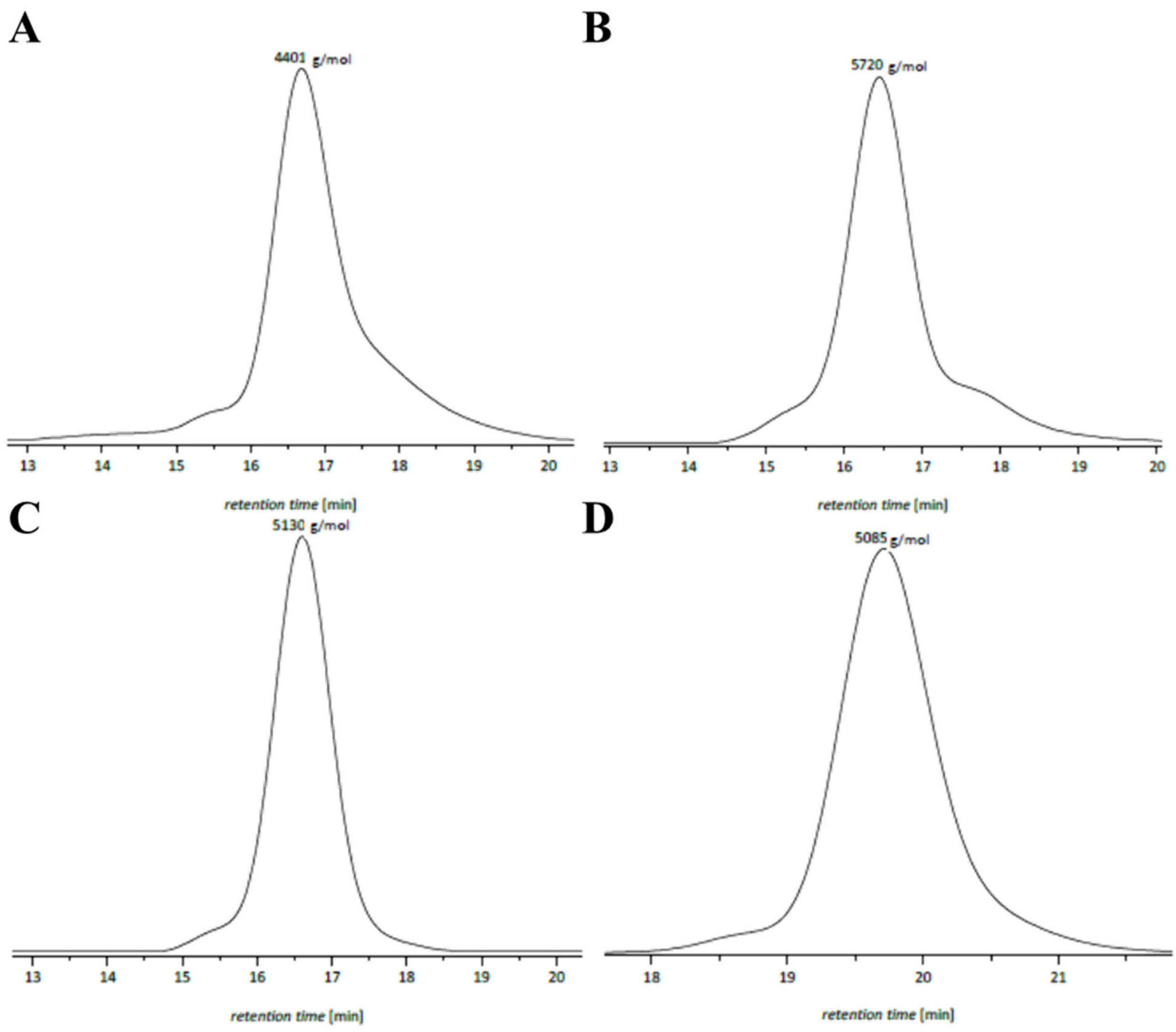

Supporting Figure 3: Size exclusion-chromatograms (SEC) of thioacetate protected A: P(MeOx), B: P(EtOx)), C: P(iPrOx), D: P(nPrOx) dithiol in DMAC.

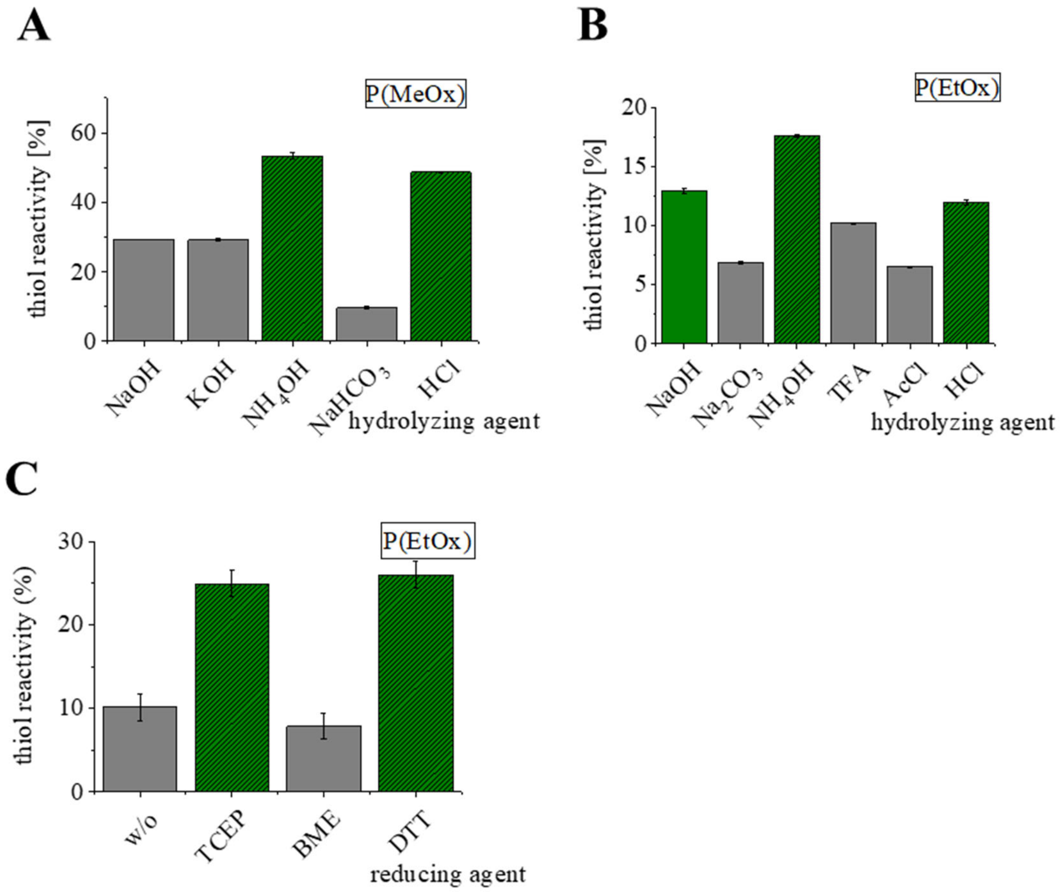

Supporting Figure 4: A: Thiol reactivity of P(MeOx) using different deacetylation reagents (green bars indicate the highest activity; striped pattern indicate comparable results to P(EtOx)), B: Thiol reactivity of P(EtOx) using different deacetylation reagents (green bars indicate the highest activity; striped pattern indicates comparable results to P(MeOx)), C: Thiol reactivity of sodium hydroxide deacetylated P(EtOx) using different reducing agents (green striped pattern indicates the highest activity).

Reactivity values were obtained before final TCEP reduction. Therefore, they are not indicative for the final reactivity given in Table 1.

Formation and mechanical characterization of POx–HEP hydrogels

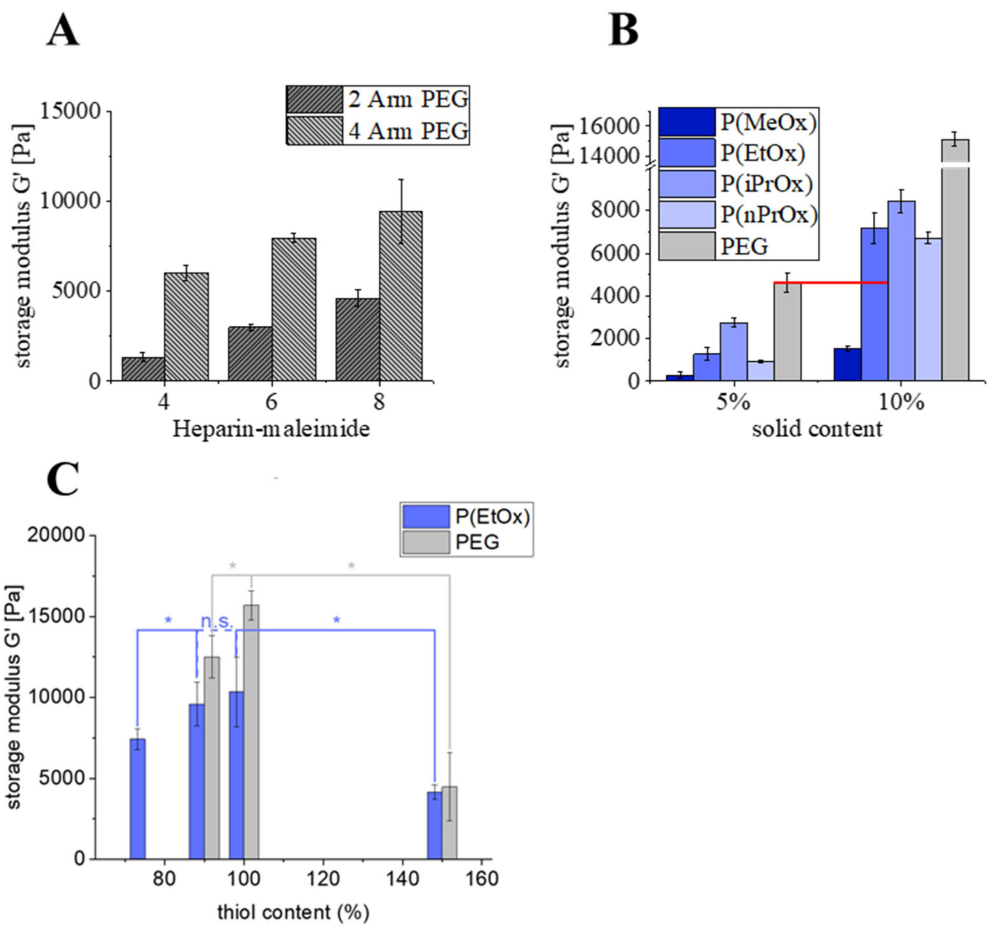

Supporting Figure 5: A: Storage moduli of 2 Arm and 4 Arm PEG-HEP hydrogels in correlation to the respective heparin maleimide used for gel formation, B: Storage moduli of POX-/PEG-HEP hydrogels formed using heparin with 8 maleimide groups in correlation to the respective solid content (the red bar symbolizes the comparable number of molecules for PEG and P(EtOx) by incorporation of their different molecular weights (44 g/mol for PEG and 98 g/mol for P(EtOx)). C: Stiffness as a function of thiol excess over maleimide groups for rating the quantitative turnover. The initially available amount of thiol groups (in percent as thiol group reactivity) was stepwise increased by adding excess of thiol group containing polymer to achieve equilibrium to available maleimide groups (100%), leading to highest stiffness. By subsequent exceeding the available maleimide groups (140%), decreased stiffness was observed, which indicates the formation of defect structures.

DSC measurements of hydrogels

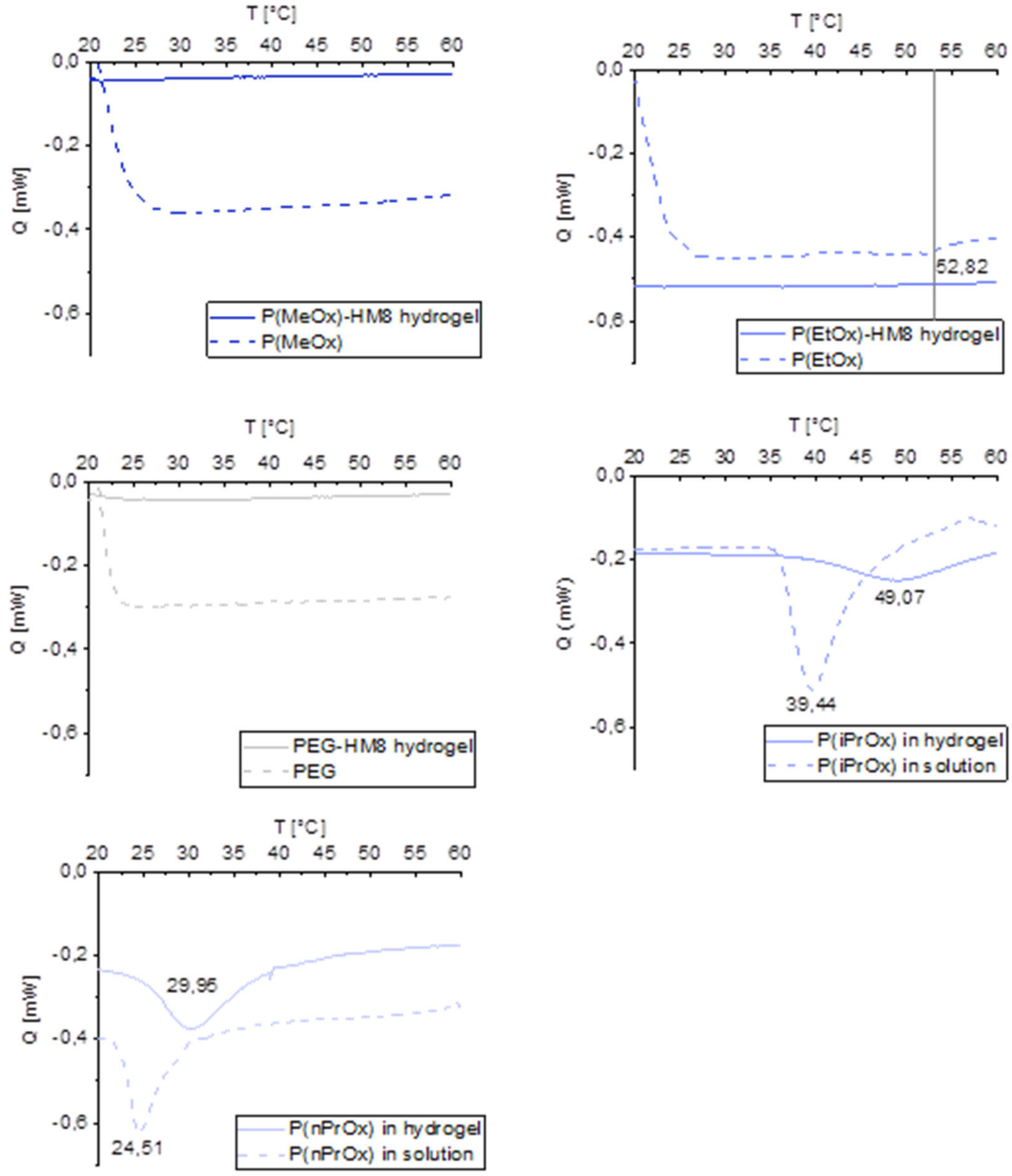

Supporting Figure 6: Calorimetric curves of POx and PEG in PBS and and as hydrogels with heparin. All samples were either dissolved in PBS or immersed in PBS; polymer concentration (PEG or POx) were 1 mg/ml.

Bioadhesion to POx-HEP hydrogels

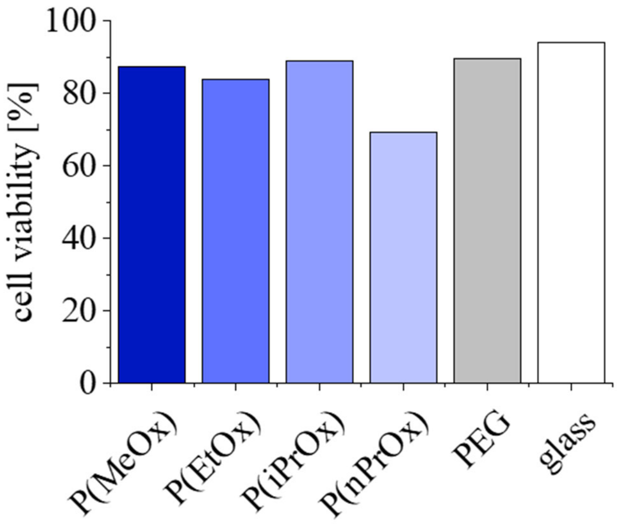

Supporting Figure 7: viability of adherent fibrobasts after 72 h adhesion on the hydrogels, determined by live/dead staining.

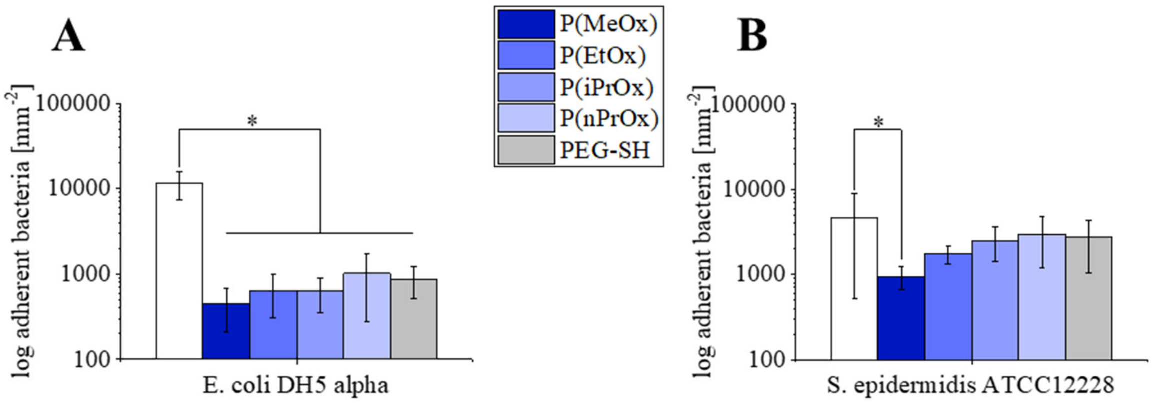

Supporting Figure 8: A: Average number of adhering *E. coli* (strain DH5 alpha) on POx-HEP and PEG-HEP layers $[(10^4\ \mu m^2)^{-1}]$, B: Average number of adhering *S. epidermidis* (strain ATCC 12228) on POx-HEP and PEG-HEP layers $[(10^4\ \mu m^2)^{-1}]$.

Hemocompatibility of POx-HEP hydrogels

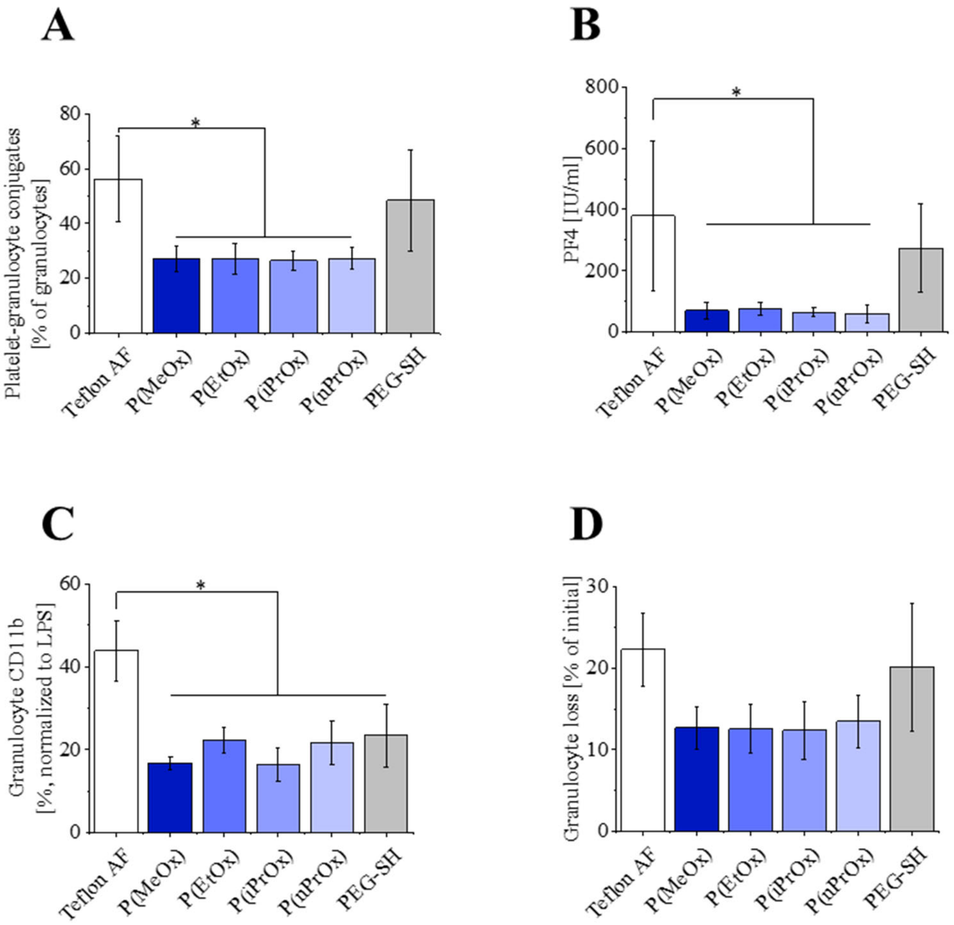

Supporting Figure 9: A: Percentage of platelet-leukocyte aggregates determined using CD41a expression in whole blood after incubation with POx-/PEG-HEP hydrogel materials, B: Concentration of PF4 (platelet factor 4) in whole blood after incubation with POx-/PEG-HEP hydrogel materials, C: Granulocyte activation determined using CD11b expression in whole blood after incubation with POx-/PEG-HEP hydrogel materials normalized to LPS control, D: Granulocyte decay in percent after whole blood incubation with POx-/PEG-HEP hydrogel materials.
